# Sensor-Generated Time Series Events: A Definition Language

**DOI:** 10.3390/s120911811

**Published:** 2012-08-29

**Authors:** Aurea Anguera, Juan A. Lara, David Lizcano, Maria Aurora Martínez, Juan Pazos

**Affiliations:** 1 Technical School of Computer Science, Universidad Politécnica de Madrid, Carretera de Valencia km 7, Madrid 28031, Spain; E-Mail: aanguera@eui.upm.es; 2 Madrid Open University, Camino de la Fonda 20, Collado Villalba, Madrid 28400, Spain; E-Mails: juanalfonso.lara@udima.es (J.A.L.); mariaaurora.martinez@udima.es (M.A.M.); 3 School of Computer Science, Universidad Politécnica de Madrid, Boadilla del Monde, Madrid 28660, Spain; E-Mail: jpazos@fi.upm.es

**Keywords:** sensors, time series, data mining, event, event definition language, stabilometry

## Abstract

There are now a great many domains where information is recorded by sensors over a limited time period or on a permanent basis. This data flow leads to sequences of data known as time series. In many domains, like seismography or medicine, time series analysis focuses on particular regions of interest, known as events, whereas the remainder of the time series contains hardly any useful information. In these domains, there is a need for mechanisms to identify and locate such events. In this paper, we propose an events definition language that is general enough to be used to easily and naturally define events in time series recorded by sensors in any domain. The proposed language has been applied to the definition of time series events generated within the branch of medicine dealing with balance-related functions in human beings. A device, called posturograph, is used to study balance-related functions. The platform has four sensors that record the pressure intensity being exerted on the platform, generating four interrelated time series. As opposed to the existing ad hoc proposals, the results confirm that the proposed language is valid, that is generally applicable and accurate, for identifying the events contained in the time series.

## 1. Introduction

There are now a great many domains where sensors record a lot of information as data streams for a set time period or on a permanent basis. Those data streams are then analysed with the aim of extracting useful knowledge from the information recorded by the sensors.

There are different approaches to analysing data in search of useful information. One approach relies on the process known as knowledge discovery in databases (KDD), which is a non-trivial process that aims to extract useful, implicit and previously unknown knowledge from large volumes of data. Data mining is a discipline that forms part of the KDD process and is related to different fields of computing, like artificial intelligence, databases or software engineering [1]. Data mining techniques can be applied to solve a wide range of problems. Techniques used to solve problems related to the study of phenomena measured with sensors in particular are very important.

There are a lot of data mining techniques and algorithms for analysing single-valued data. However, domains where large volumes of data are generated and recorded by sensors as continuous streams, such as measurements by seismographs, patient monitoring devices or fire detection mechanisms, are increasingly common. This type of data, called time series, have peculiarities whose analysis requires specialized techniques.

Formally, a time series can be defined as a sequence *TS* of time-ordered data *TS* = {*TS_t_, t* = 1,…,*N*}, where *t* represents time, *N* is the number of observations made during that time period and *TS_t_* is the value measured at time instant *t*. In some domains, the value of not one but several observations might be measured at each time point, leading to multidimensional time series.

There are many well-known problems in time series analysis and different data mining techniques to solve them. Most time series analysis techniques consider whole time series [2,3]. However, there are many problems where it is requisite to focus on certain regions of interest, known as events, rather than analysing the whole time series [4]. This applies in areas concerned with analysing momentary events. One example is seismography, where the points of interest occur when the time series shows an earthquake, volcanic activity leading up to the earthquake or replications.

From the viewpoint of information theory, the concept of time series event is closely related to the concept of entropy [5,6]. System entropy means the amount of information contained in a set of system symbols. In our case, the systems are time series, and the events are regions of the series that contain more information, that is, that have greater entropy.

**Figure 1.**
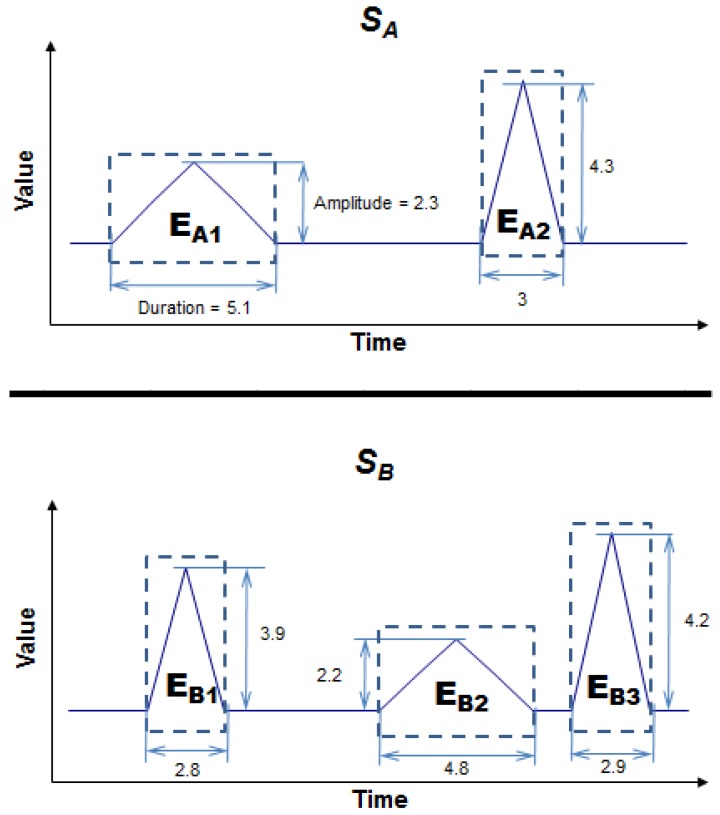
Charts showing two time series and event features.

The conception of what an event is varies from domain to domain. Suppose, for instance, that the events of the time series in a particular domain are the peaks generated by the local maxima. Given two time series, *S_A_* and *S_B_* (Figure 1), there are two regions of interest in series *S_A_* and three in series *S_B_*. Let us assume that the interesting features of the events in this particular domain are Duration and Amplitude. Comparing the two series, we find that the first event in *S_A_* (*EA_1_*) is very like the second in *S_B_* (*EB_2_*) because both events have a similar duration and amplitude. The third event in *S_B_* (*EB_3_*) is also very like the second in *S_A_* (*EA_2_*). In this case, series *S_A_* and *S_B_* have two events in common and are, therefore, very alike.

To be able to extract useful knowledge from time series containing events, it is necessary to first identify those events, as they are the only regions of the time series that provide information of interest. For example, to extract conclusions about the characteristics of seismographic phenomena in a particular geographical region, we will have to analyse those instants recorded by the seismograph that match up with the occurrence of such phenomena, as the information recorded in the remainder, when there is no seismic activity at all, is of no interest to the expert. The identification of such events is an open problem. Most existing techniques do not solve this problem: they are either only applicable in particular domains for which they propose ad hoc mechanisms or they propose the identification of meaningless landmarks of the series that are as they do not include any expert knowledge. To solve this problem, we propose an events definition language for multi-dimensional time series. This language is designed to be general enough for application in any domain. After the events have been defined, a translation tool designed as part of this research automatically generates source code in a high-level language that can be instantiated by specific time series, returning a list of events that they contain. This tool manages to reduce the dimensionality of the series whereby they can be converted into a sequence of events that make sense to the expert. To test the validity of this language, it was applied to time series generated in the medical field of stabilometry. Stabilometry is a discipline looking into human balance. A device, called posturograph, is used to study balance-related functions. The patient stands on a platform and completes a series of tests. We used a static Balance Master posturograph. In a static posturograph, the platform on which the patient stands does not move. The platform has four sensors, one at each of the four corners: right-front (*RF*), left-front (*LF*), right-rear (*RR*) and left-rear (*LR*). While the patient is completing a test, these sensors record the pressure intensity being exerted on the platform, generating four interrelated time series.

The proposed language, which will be described in Section 3, will be applied to this type of time series. The results of applying the language will be discussed in Section 4, whereas the findings and future work will be detailed in Section 5. Beforehand, we will present work related to this article in Section 2.

## 2. State of the Art

**Figure 2.**
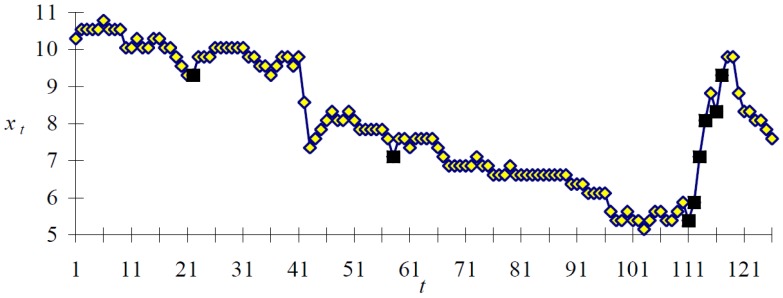
Financial time series.

In this section, we describe techniques concerned with time series events analysis, where events mean regions of a time series that provide meaningful information. One of the first proposals for analysing time series events was presented in [7], describing a framework called TSDM. TSDM was based on the definition of an events characterization function to determine how far ahead an event can be forecast. Clustering techniques are used as a tool for grouping event forecasting patterns. The method was applied to financial time series with the aim of forecasting when there will be a steep increase in the value of a company on the stock exchange, as there is an interesting buying opportunity just before the increase. Figure 2 shows a time series representing a stock exchange price. The diamonds present the stock exchange value, whereas the boxes denote buying opportunities.

Other proposals, like [8], resemble the above technique, except that, in this case, there is *a priori* no knowledge on the form of the patterns preceding an event. This technique also proposes a way of identifying events. By applying a function of interest to each possible subsequence of the time series, only subsequences obtaining a value of interest greater than a certain threshold are considered as events. This process, however, looks to be too costly from the computational viewpoint, as a window would have to move over the time series and evaluate the function of interest for each subsequence. Additionally, this process would have to be repeated for all possible window sizes, as, generally, the events duration is not known beforehand.

Event identification is one of the major challenges, as they are the only time series regions that provide information of interest from which the expert can to extract useful knowledge. There are proposals in this respect. Guranik and Srivastava [9] propose dividing the time series into subsequences, which are further divided until they meet a particular stopping condition. The basic idea of this proposal is to divide the time series using change points, that is, points at which the behaviour of the time series changes.

The technique described in [10] has some similarities with the above in the sense that it is based on analysing the time series landmarks, which are the maximum, minimums and break points. They propose a procedure called *Minimal Distance/Percentage Principle* (MDPP) to smooth the curves, storing only 1.5% of the original data of the time series, and with an error of only 7.5%. This smoothing procedure is based on the idea of removing the landmarks when they are very close to each other on both the x- and y-axis. They consider the use of a series of transformations (amplitude, time warping,…) that they apply to the landmarks. The technique proposes to store characteristics of the maximums and minimums of the curve. Several of these characteristics are invariant when some transformations are applied, and only the invariant characteristics are taken into account when searching similar series under particular transformations. For example, if the time warping transformation is applied to the landmarks of two series and the distance after that transformation is less than a particular threshold, we can conclude that those two series are similar but one is compressed with respect to another, that is, if we temporally compress one of the series, we get the other or a similar series.

The above two techniques have a drawback; they do not use expert knowledge to determine the regions of interest of the series but are based on the analysis of certain landmarks of the series like the extreme points or break points. These proposals are insufficient as, in many domains, the conditions for determining events go beyond the mere identification of a landmark.

Another method is proposed in [11] to build reference models from heterogeneous time series, that is, the magnitude measured in each time series does not necessarily have to be the same. This is a very versatile scheme but also has the drawback of requiring a previous step to transform the time series to the same magnitude. The method is based on the previous transformation of the time series to a set of events, which are subsequences of the time series that meet certain conditions. The process of determining these conditions is highly dependent on the domain in question and many low-level changes are required to move from one domain to another. Once the time series have been divided into events sequences, they are analysed using artificial intelligence techniques, known as interval temporal logic (ITL).

ITL is based on defining relationships among temporal intervals (*precede, coincide, contain, etc.*). An algorithm using ITL elements is defined to extract associations among the different events to determine, for example, what events precede others, what events coincide with others, *etc.* The technique has been evaluated with time series that measure wind speed and air pressure in Beijing. Interesting temporal patterns relating both magnitudes were obtained after applying the technique to those data.

The events definition language proposed as result of our research aims to solve the limitations of the above approaches that focus solely on one-dimensional time series analysis and are insufficient for identifying events in any other domain because they are not very portable, they do to use expert knowledge necessary to correctly define what an event is in each domain, and are not applicable to multidimensional time series.

## 3. The Proposed Solution: An Events Definition Language in Time Series

There are few proposals in the literature that deal with the problem of event identification. In actual fact, methods usually divide a time series into subsequences based mainly on statistical concepts such as the change point. Many such techniques are not general enough for use in different domains as each domain is different to the others and therefore the conditions determining whether something is or is not an event are particular to each domain.

This is compounded by the circumstance that most techniques deal with events in one-dimensional time series, obviating the complexity arising when we have multidimensional time series, as there may be dependencies among the different attributes (dimensions) of the time series.

To overcome the weaknesses of existing techniques, which may not be general enough for use in different domains and with multidimensional time series, we have proposed a formal events definition language enabling domain users to define events in domain time series. Apart from that language, we have developed a translator that checks whether an events definition obeys the language rules and is lexically, syntactically and semantically correct. Otherwise, errors are reported to the user. When the events definition is correct, it is translated to source code in a high-level language, automatically generating a module that contains the source code to identify the events in the time series of the respective domain. This module provides a method that outputs the events identified in the time series that it receives as a parameter. In this section, we give an overview of the proposed language and the developed translation tool.

### 3.1. Language Description

To specify the events definition language, we have followed a process that aims to simulate how a human being visualizing a time series naturally locates events. When identifying events, people intuitively try to locate points in the series that have certain particularities. Generally, these are points at which the series takes an outlier value in relation to the other points of the series. These points are usually maximums, minimums, points at which the series intersects a particular value, *etc.* Sometimes, these points of interest have to be filtered to determine which are really interesting and which do not meet the necessary conditions to be so. After locating the points of interest, human beings try to search backwards in the series for the point that meets a particular condition and determines the start of the event. Similarly, they search forward in the series to determine where the event ends. Normally these start and end points are points at which the series is or returns to normal before and after the occurrence of the event.

The proposed language uses basic concepts of set theory, logic, algebra and descriptive statistics. Set theory is necessary as the points of interest of the series are located by means of a process of refining more general sets of points as described earlier. Logic is required to establish the conditions that determine what really is a point of interest in the series and what conditions such points have to meet to be part of an event. Algebra plays a fundamental role as arithmetic operations are needed to perform calculations on sets of points of interest in the series and their respective value. Finally, descriptive statistics is used to represent the normality or stability of the series, where such normality is represented by means of descriptive measures of centralization and dispersion, such as the mean, mode or standard deviation. These measures are used to determine regions of the series that are removed from normality and have high entropy and which, therefore, are potential events.

From the formal viewpoint, the proposed language uses a ALFEUC-type description logic (according to the usual naming convention based on labels) [12], that is, a description logic with a base language (AL) that models concepts, roles and individuals and also includes functional properties (F) to characterize events using descriptive statistics, full existential qualification (E) to algebraically model the comparison against models or patterns and concept union (U) and negation (C) to process time series according to set theory. Some of the elements of the language are predefined and others will have to be defined for each particular domain. The aim is to offer a simple language that is comprehensive enough to be generally applicable. The events definition procedure is as follows:

1. Define the set of points of interest in the time series, which will be the basis for defining events. Note that these sets refer to time instants (timestamps) and not to the value that the time series takes at that time instant. Therefore, these sets will be ordered, will not contain repeated elements and will include only positive integer values.
2. Use of the basic language elements and the sets of points of interest to define the events. We have devised a series of basic predefined elements for the events definition language that will usable in any domain and should be helpful to users who will not have to define such elements. These elements are outlined in Table 1.

The language also includes other basic elements present in any language, such as, for example, identifiers, numerical and logical constants, *etc.* The body of each events definition is delimited by braces and preceded by the reserved word *def*. The *def* body contains the three types of elements described below. An events definition can include any number of elements provided that they are arranged in the following order.

#### 3.1.1. Statement of Time Series

The time series can be either multidimensional or one-dimensional, but only real number series are considered (if they were integer value series, all the values would be transformed to real numbers). To define a time series, we use the reserved word *series* and, then, we specify the name of the time series. A statement can include any number of time series (at least one), where each statement is separated by a semicolon (;). The first two rows of Table 2 specify the details of the syntax for stating time series together with several examples.

No two time series can have the same name. Once a time series has been defined, it will then be able to be used for the definition of statistics and basic sets on the time series. Additionally, time series will also be able to be used as part of an arithmetical expression.

#### 3.1.2. Definition of Points of Interest/Landmarks

The sets of points of interest contain timestamps that the domain expert considers to be relevant and are the basis for later defining events. A set of points of interest is always defined from a previously defined or predefined set. This simplifies the expert's job. There are two options:

- **Condition-based definition.** In this case, sets can be defined based on the elements of pre-existing sets by filtering with a condition. To do this, we use the notation described in Table 2. The *set_name*′ identifier must correspond to a previously defined (predefined or derived) set. The predefined sets of the language include the set *max*, which contains all the timestamps of a time series in which there exists a local maximum, and the set *min*, which contains all the timestamps of a time series in which there is a local minimum. The scope of the *id_1_* identifier is confined to the braces that are used to define the set.
  Table 2 includes an example of a set called *MaxMultiples50* composed of the all the timestamps of the time series *TS_A_* where there is a local maximum and that are multiples of 50.
- **Operator-based definition.** In this case, a set is defined from another two sets defined previously using the union, intersection and difference operations. The notation used is again listed in Table 2. In this case, *new_set* is the name of the new set that is to be defined and *old_set_1_* and *old_set_2_* are previously defined sets. The names of both *old_set_1_* and *old_set_2_* have to be different than the name of the *new_set*. On the other hand, OPERATOR can take the values of *joint* (union), *intersec* (intersection) or *diff* (difference). Although it is perfectly possible to define these sets using conditions, we have decided to include these operator-based mechanisms of definition to make things easier for users. Table 2 includes several examples of the definition of sets using the above operators.

No two sets can have the same name. Once a set has been defined, it can then be used to define other sets and to define events. A definition can include any number of sets (even none), where each definition is separated by a semicolon (;).

#### 3.1.3. Definition of Events Proper

An event type is always defined using points of previously defined sets, and an event break point, plus the event start and end points, are determined. The events definition notation is listed in the bottom two rows of Table 2. The *set_name, set_name_1_* and *set_name_2_* identifiers must correspond to the previously defined sets. These can be the same or different sets. The scope of the *break_point* is confined to the braces used to define the events.

The bottom row in Table 2 includes an events definition example that uses the set *MaxMultiples50* defined earlier in Table 2. Suppose that, in a particular domain, the points of this set are the break points and that the events start at a timestamp before and end at a timestamp after these break points. Considering that *previous* and *next* are two predefined language operators (respectively returning the previous and next element to one given in a particular set), this type of events are defined as illustrated in the bottom row of Table 2.

#### 3.1.4. Definition of Conditions and Expressions

The conditions used to define sets of points and events can be built from expressions and relational and set operators. Additionally, compound conditions can also be defined using the logic operators *and* (‘&&’), *or* (‘‖’) and negation (‘!’). Moreover, the expressions can be simple or compound. The simple expressions can be integer, real or Boolean. A simple expression can be:

- An identifier
- An integer constant
- A real constant
- A Boolean constant (*true* or *false*)
- The start of an event. This is a special type of identifier that must always have the same name. For this purpose, we use the reserved word *start*.
- The end of an event. This is a special type of identifier that must always have the same name. For this purpose, we use the reserved word *end*.
- The break point of an event. This is a special type of identifier that must always have the same name. For this purpose, we use the reserved word *break_point*.

It is also possible to define compound expressions, making use of the different arithmetic operators, the time series access operators and the set operators.

Even though the events definition language is very rich, there can be circumstances in which it is very hard to express some sort of special type of specific operation in any particular domain. To solve any such exceptional problem, the language provides for a mechanism whereby the expert, with the help of a programming literate assistant, can define functions in a high-level language that will be rendered just so in the code generation process.

These *embedded functions* will have been defined at the start of the file (before defining the time series), specifying their name, output value type, number and type of parameters and including the code in the respective high-level language (between the special characters /@ and @/). The syntax is as follows:

extmethod name output_type parameter_no parameter_type*
/@
HIGH-LEVEL LANGUAGE TYPE
@/
;

That is, we use the reserved word *extmethod*, the function name, output type, parameter number, parameter type and the method code between special characters. We can define as many functions as we like, each separated by a semicolon. The admissible types for the output value are *bool, int, float* and *void*. The admissible types for the input parameters are *bool, int* and *float*. The function name and types have to match the function name and types defined between the special characters (/@ and @/) in the respective high-level language, although this is the language user's responsibility.

We give several examples of the use of the events definition language in time series later. For other additional examples, as well as an exhaustive description of the language elements and the rules governing events definitions, see [13].

### 3.2. Translation of Events Definition to Source Code in a High-Level Language

The proposed language enables domain users to formally define domain events straightforwardly. We have also developed a translator that is able to check that the definition made by the user is lexically, syntactically and semantically correct. If this definition is not correct, it reports the errors to the user. If, on the other hand, the definition is correct, it automatically generates a software module that contains source code in a high-level language identifying the time series events in the respective domain. To simplify the integration of the events identification software module, it has been translated to the high-level language C#, as this is the language used to develop the time series analysis applications used by the experts of the different domains that have collaborated in this research. The events identification module provides a method that outputs the events found in the time series. An events definition file can define several types of events, and, therefore, the events identification module has been designed to generate a list that orders events temporally. Each of these lists is given the name that has been assigned to the events type in the events definition file. Additionally, each event is characterized by its start and end points and its break point. The events identification module has an interface for instantiating specific time series. To do this, this module supplies a method that receives the following parameters:

- Time series size.
- Defined time series. Each time series can be seen as a list or vector of numbers. The name of each time series will have to match the name of the time series stated in the file that contains the events definition.

**Figure 3.**
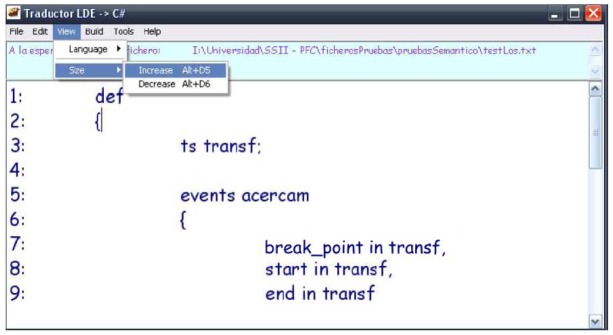
Edition and translation environment.

In order to translate a particular events definition to source code in a high-level language, we have developed an application that is capable of editing events definitions, checking their lexical, syntactic and semantic correctness and, finally, translating that events definition to source code in a high-level language. Figure 3 illustrates the interface of this multi-language application, showing the tool menus at the top and the editable area with the events definition immediately below.

A series of video tutorials are available on the YouTube platform, each detailing how to use part of the application and the data that it outputs. The video tutorials are currently available in Spanish, as this is the language of the experts now using the system (video tutorials in English are to be made available to experts in the next phase):

- Editing events definition files (http://www.youtube.com/watch?v=0tdsPLmbEWA)
- Correcting errors (http://www.youtube.com/watch?v=v-DQeWY4YVM)
- Using generated source files (http://www.youtube.com/watch?v=NKcRfZJO61U)

As mentioned, the above tool enacts a compiler-like translation process, executing different lexical, syntactical and semantic analysis and code generation phases. In the following, we describe the design decisions taken regarding the events definition language translator for each of the above phases (for a more detailed description of translation tool design and implementation issues, see [13,14]).

#### 3.2.1. Lexical Analysis

We have designed and implemented a lexical analyser to be able to check the lexical correctness of an events definition. The smallest meaningful language elements (*tokens*) are as follows:

- **<ID, ptr(ST)>** 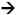 Identifier. In this *token, ptr(TS)* is the pointer to the respective identifier in the symbol table.
- **<PR, ptr(ST)>** 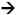 Reserved Word. In this *token, ptr(TS)* is the pointer to the reserved word in the symbol table.
- **<INT, num>** 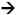 Integer constant. For this token, *num* is the numerical value of the integer constant.
- **<REAL, num>** 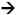 Real constant. For this token, *num* is the numerical value of the real constant.
- **<BRACE_O,->** 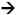 Opening brace (‘{’).
- **<BRACE_C,->** 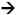 Closing brace (‘}’).
- **<SC,->** 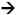 Semicolon (‘;’).
- **<PAR_O,->** 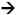 Opening parenthesis (‘(’).
- **<PAR_C,->** 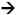 Closing parenthesis (‘)’).
- **<COMMA,->** 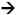 Comma(‘,’).
- **<AOP, n>** 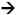 Arithmetical operator (*n* is the operator code, as set out in Table 3).
- **<ROP, n>** 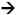 Relational operator (ROP operator code in Table 4).
- **<CMDOP, n>** 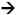 Conditional operator (CMDOP operator code in Table 5).
- **<TSAOP, n>** 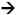 Time series access operator (TSAOP operator code in Table 6).
- **<SETOP, n>** 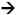 Set operator (SETOP operator code in Table 7).
- **<FUNCTION, code>** 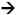 External function. This *token* represents the source code of a high-level function defined in C#. These functions are a resource for use in circumstances where it is very hard to express some sort of special type of operation with the proposed language. A function is not considered as a *token* in a high-level language. In this case, however, it is a smallest meaningful element that can be seen as a character string that will be rendered unchanged in the code generation process.

Tables 3 to 7 list the different types of language operators and their associated numerical code.

To recognize the language tokens, we have built a regular grammar and a deterministic finite automaton, which is extended with a series of semantic actions. Both are detailed in the following.

The lexical analyser grammar productions for the events definition language are as follows:

A 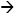 l′K | dN | { | } | ( | ) | ; | , | + | - | * | % | /C | = P | !Q | <R | >S | &T | |U | fV | delA | .Z
K 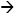 lK | dK | λ
C 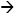 / D | @EM | λ
D 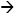 c′D | CAR A
EM 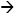 c″EM | @EM′
EM′ 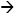 /
N 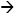 dN | .F | λ
F 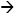 dF_1_
F_1_ 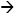 dF_1_ | λ
P 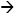 = | λ
Q 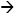 = | λ
R 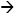 = | λ
S 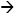 = | λ
T 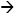 &
U 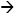 |
V 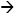 ′V_1_ | lK | dK | λ
V_1_ 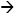 ′ | λ
Z 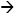 vZ_1_
Z_1_ 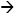 aZ_2_
Z_2_ 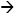 lZ_3_
Z_3_ 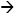 uZ_4_
Z_4_ 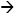 e

In this grammar, the axiom is *A*, and the non-terminals are *A, K, C, D, EM, EM*′, *N, F, F_1_, P, Q, R, S, T, U, V, V_1_*, Z, Z_1_, Z_2_, Z_3_ and Z_4_.

The other symbols are terminals. There follows a description of some special terms:

*l′* = any letter except ‘*f*’
*l* = any letter (*a-Z*)
*d* = any digit (0–9)
*del* = delimiter (space, tabulator, carriage return)
*c′* = any character except ‘*CR*’ (carriage return)
*c″* = any character except at ‘@’
*CAR* = carriage return

**Figure 4.**
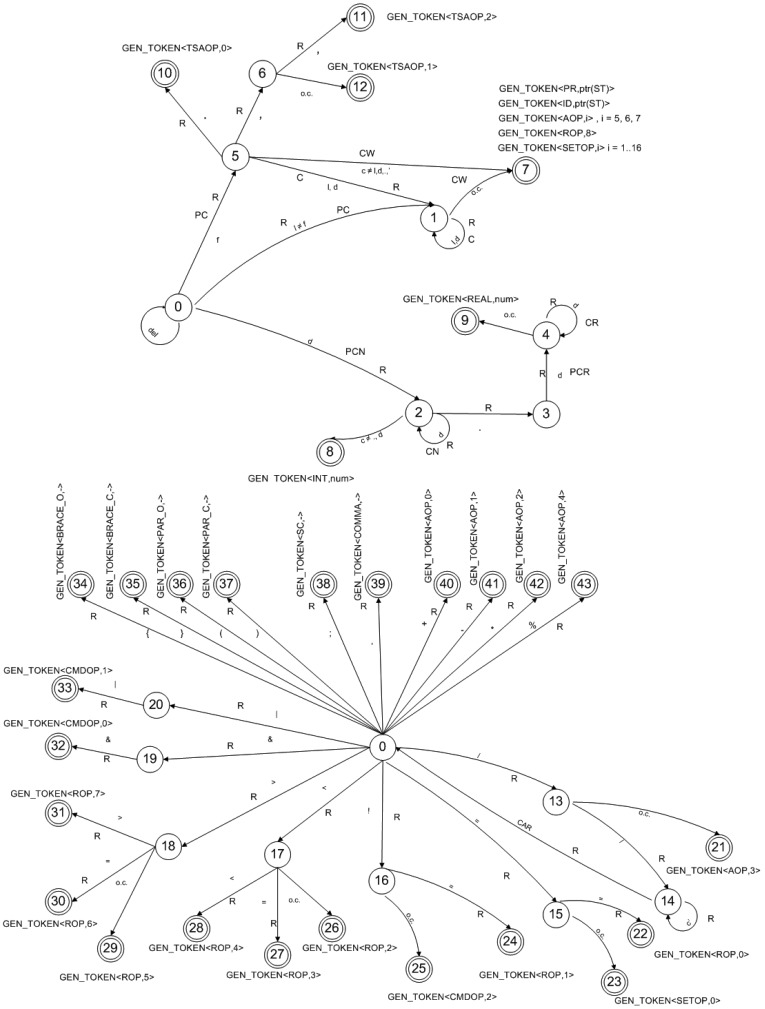
Lexical Analyser Automaton.

Figure 4 shows the deterministic finite automaton proposed for the time series events definition language.

The automaton includes several semantic actions, which are described below:

- **Read (R)**: Reads an input file character.
- **PreConcat (PC)**: Action that is executed when the first character of an identifier is read. It places the read character in the first position of the global *id* variable and sets its *length* to one, where *length* is a variable parameter indicating the maximum possible identifier length.
- **Concat (C)**: Operating like PreConcat, it inserts the read character in the global *id* variable in the relative position indicated by the *length* variable, increasing this variable by one unit.
- **Classify_Word (CW)**: An action executed when a lexeme has been received. The mission of this action is to determine whether the received lexeme is a reserved word, an operator, an identifier already existing in the symbol table or a new identifier. To do this, it searches a table that contains the language operators composed of letters (*abs, exp, sqrt, etc.*) for the read lexeme. If it finds the lexeme, it generates the respective token. If it is not an operator, CW will search the symbol table to find out whether it is a reserved word. If it is, CW will generate the respective token. If it is not a reserved word, it is an identifier and, therefore, CW checks that it does not exceed the length limit. If the length is correct, CW checks whether the identifier was already in the symbol table, and if not, it inserts the identifier. Finally, CW uses the Generate token semantic action to generate the respective token with the symbol table address of the identifier or reserved word (as applicable). We have used a hash table to implement the symbol table to assure search efficiency.
- **PreCalculateNum (PCN)**: Action that assigns the *number* variable to the numerical value associated with the read character.
- **CalculateNum (CN)**: Action used to add the read digit to the number that we have processed. To do this, it multiplies the numerical value of the read digit by 10 and adds it to the *number* variable that stores the value of the number read so far.
- **PreCalculateReal (PCR)**: Action that is executed when reading the first digit of the decimal part of a number. The value 1 is assigned to the *digit_position* variable. Then the read digit is divided by the value (10* *digit_position*) and the result is added to the *number* variable which has been generated while the integer part of the number was read.
- **CalculateReal (CR)**: Action used to add the read digit to the number that we have processed. To do this, it increments the *digit_position* variable by one unit. Then it divides the read digit by the value (10* *digit_position*) and adds the result to the sum of the *number* variable that accumulates the value of the number read so far.
- **ReadFunction (RF)**: Action that executes while the C# code of an external function is being read. When the first character is read, its length is initialized to 1 and that character is stored in the first *code* position. For the following characters, this action stores the character read at the end of the *code* variable and increments its length by one unit.
- **GenerateToken (GEN_TOKEN <-,->)**: This action receives one or two parameters and generates the respective token accordingly. It checks whether it fits into the two reserved octets (less than 2^16^) if it is an integer or into four octets if it is a real number. No further checks are run for other non-numerical tokens.

#### 3.2.2. Syntactic Analysis

We have designed and implemented a syntactic analyser to check the syntactic correctness of an events definition. This syntactic analyser is based on a free context grammar that includes the developed events definition language syntax. The details of the grammar are shown in Table 8.

#### 3.2.3. Semantic Analysis

The semantic analyser is the last of the three input file analysis stages to be carried out. The goal of this analysis is to capture any user errors that the lexical and syntactic analysers are unable to detect.

This analyser breaks with the idea of a standard scheme such as is used in the syntactic analyser. The actions that it carries out are tailored for each grammar. Even so, there is a strong relationship between the two, as they share data structures, and the syntactic analyser controls the execution of the semantic analyser.

All the logic of the semantic analyser is located in simple actions called *semantic actions*. These actions are a set of instructions that are always executed under the same scenario, associated with the reduction of a syntactic grammar rule. For example, the semantic action *A* 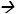 *B* = *C {Action1}* indicates that *Action1* will be executed when the syntactic analyser carries out a reduction applying rule *A* 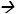 *B* = *C*.

The semantic analyser is mainly concerned with type analysis throughout the file, although, depending on the programmer and the design of the other two analysers, it may have a number of other functionalities.

For all the details on the semantic actions designed and implemented for the events language translator, see [14].

#### 3.2.4. Code Generator

The translator is the part of the tool responsible for generating object code written in a high-level language from the data structures received from the above three analysers.

The translation generates three output files: a file containing some predefined language functions, a file containing embedded functions and a file with the translation of the events definition to a high-level language. Each file contains the following elements:

- **Predefined Functions File.** Any predefined function or functions (max, min, mean, stdev, variance, mode, median, exp, sqrt, subc, subcp, supc, supcp, isfirst, islast, in, joint, diff, intersec, next or previous) used by the user in the source code will be loaded just once in this file.
- **Embedded Functions File.** This file stores the code of the functions written in a high-level language that the user has added as an extension to the events definition.
- **Translation File.** This file contains the results of the translation of the source code to the high-level language, making public the events identification operations on real time series that are passed as parameters.

**Figure 5.**
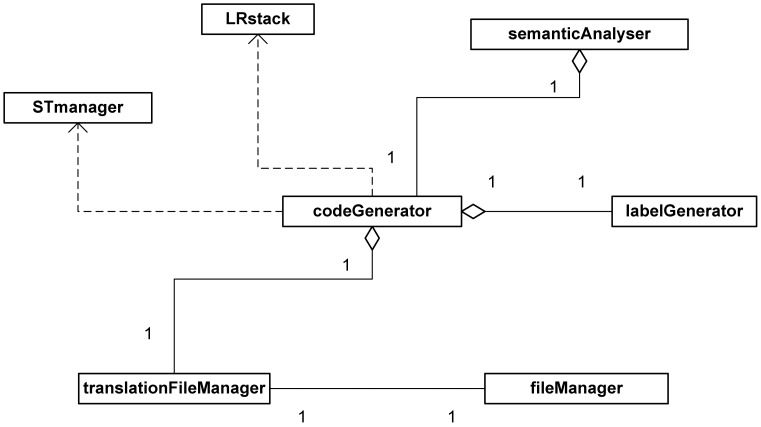
Simplified code generator class diagram.

Figure 5 shows a class design diagram for the subsystem implementing the application's translation module. This module (class *codeGenerator*), combined with the semantic analyser (class *semanticAnalyser*), uses the syntactic analysis stack (class *LRstack*) and the symbol table (class *STmanager*) to translate the input file managed by the respective module (classes *translationFileManager* and *fileManager*). The class *labelGenerator* is a module used to improve the usability of our system including labels in different languages (English and Spanish) to adapt to users' native language.

## 4. Case Study: Stabilometry Experimentation

Balance disorders and vertigo are two of the most common diseases that physicians come up against every day. About 30% of the population will have experienced an episode of vertigo before they reach the age of 65. As of 65 years, this type of disease becomes much more prevalent, and is considered as the primary cause of falls in elderly people.

**Figure 6.**
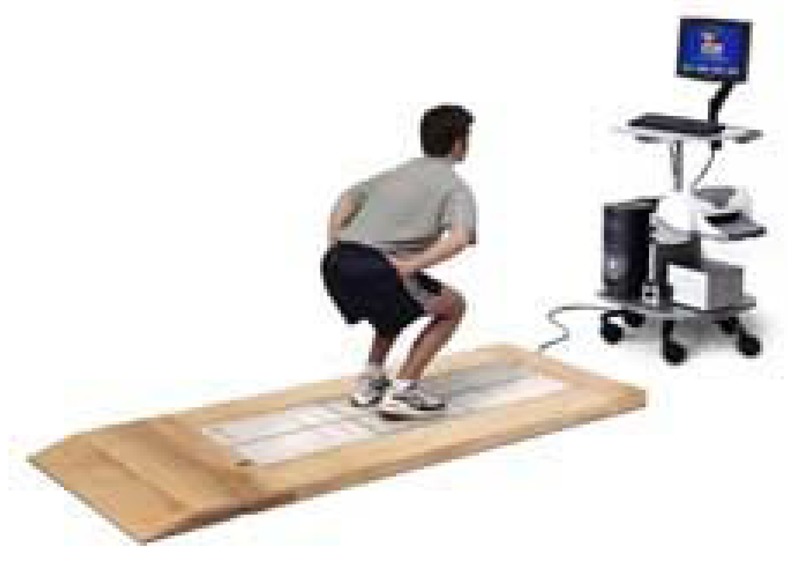
Patient completing a test on a stabilometric platform.

Stabilometry is a set of techniques that analyse people's postural control [15,16]. It is also known as posturography, statokinesiometry and posturometry. To gather information about postural control, stabilometry is based on the use of dynamometric platforms, sensitive to the horizontal and vertical forces to which they are subjected. These forces are recorded by sensors appropriately situated on the platforms that are then connected to software systems capable of visualizing the position of the subject's centre of gravity. Figure 6 shows a patient performing a test on a stabilometric platform.

Stabilometry dates back to around 1850, when Romberg [17] conducted some studies to check the sway of individuals with their eyes open and closed, and Bárány (Nobel Prize in Medicine) described postural instability and explored the vestibular-spinal function in patients with vestibular injuries [18]. The birth of stabilometry in the late 19th century led to the development of a number of postural control techniques that were very uncomfortable and invasive for the patient. Hence, a second current of thought that set out to record sway by analysing the pressure exercised by the subject on a platform. This tendency culminated with Baron's statokinesiometer [19], a very popular posturographic system composed of four electromagnetic pressure sensors. This device can be considered to be the forerunner of today's stabilometric systems.

Postural control is a key element for understanding a person's ability to perform their routine activities. The aim is to maintain body equilibrium, either at rest (static equilibrium) or in motion or subject to a range of stimuli (dynamic equilibrium). There are two main objectives:

- Balance, that is, the ability to maintain projections of the centre of gravity within the base of support.
- Posture, that is, the ability to keep the different parts of the body correctly aligned with each other and the surrounding environment.

To measure postural control, patients take a series of test. The different tests are designed to single out the principal sensory, motor and biomechanical components of balance with the aim of being able to assess an individual's ability to use them individually or jointly [20].

Although stabilometry was originally devised merely as a patient postural control and balance assessment technique, it is now considered to be a useful tool for diagnosing ([21,22]) and treating balance-related disorders [23]. Some examples of its use are:

- Analysis of the influence of age and sex on postural control, primarily the study of balance loss in elderly people and people with motor diseases [24–27].
- Balance analysis in patients with neurological diseases [21,28].
- Study of the effect of different drugs on balance [29].

Modern posturography is divided into two major types:

- Static posturography: uses fixed platforms for measuring patient sway through the pressure exerted by their feet on the platform.
- Dynamic posturography: is based on the use of a platform placed on a horizontally movable support, which inclines forward or backward and rotates around an axis that is collinear with the ankles. One of the best-known dynamic posturography systems was developed by Nashner ([30]) and then studied by Black [31,32].

Throughout this research, we have used a modern static posturography device called Balance Master, manufactured by NeuroCom^®^ International [33]. Previous research has demonstrated its accuracy and reliability for postural control analysis [34,35].

The most renowned hospitals and reputable medical and sports institutions own a Balance Master or another device manufactured by the same company nowadays. This type of device is used in different fields, including ear, nose and throat specialities, neurology, psychiatry, geriatrics or sports medicine. Although it is better known and more popular in the United States, the use of this type of devices is a challenge for medical and sports institutions in Europe and especially Spain.

Balance Master is a simple but very powerful device. It is composed of a metal plate placed on the floor and divided into two interconnected longitudinal plates. The metal plate is surrounded by a wooden platform, whose only mission is to prevent patients from stumbling and falling. The patient stands on the metal plate and completes different types of tests. The patient has to complete the tests in a particular order following the physician's instructions. The set of tests completed by the patient in a session is composed of what is called an assessment protocol. The aim of each assessment protocol is to measure different parameters related to patient balance. Additionally, each assessment includes trials, including minor variations. Finally, each trial is repeated several times.

**Figure 7.**
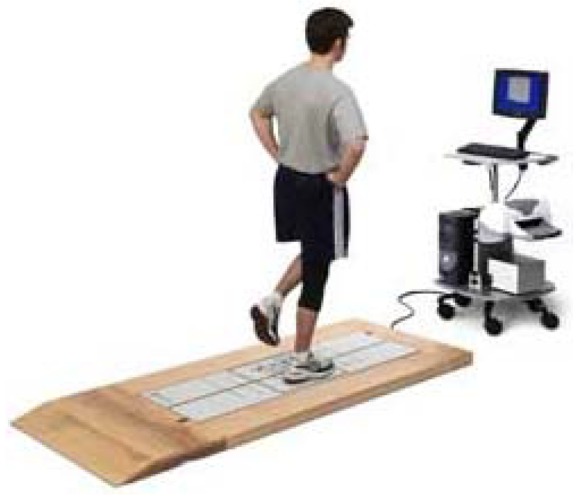
Patient completing the US assessment protocol with right foot on platform and eyes open.

**Figure 8.**
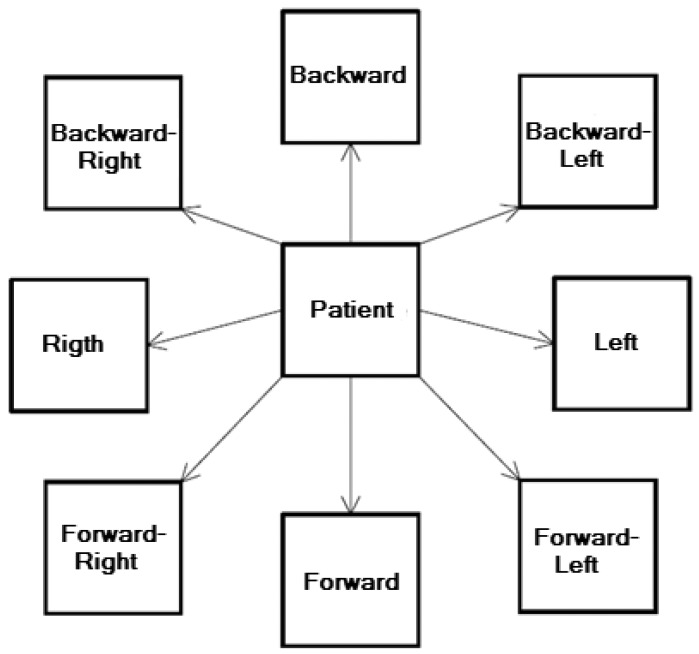
Target positions towards which patients have to displace their centre of gravity in the LOS assessment protocol.

**Figure 9.**
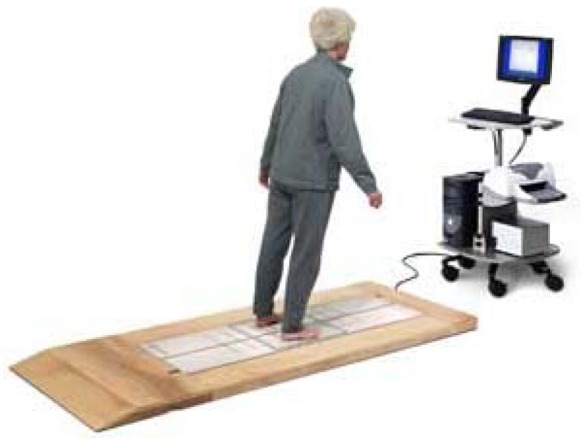
LOS assessment protocol performance with forward displacement.

**Figure 10.**
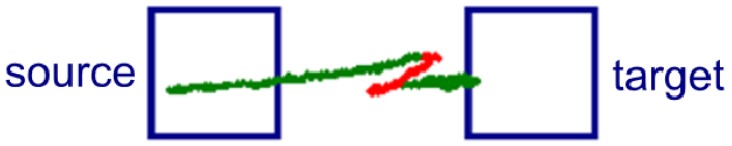
Example of the patient trajectory during the LOS assessment protocol Right trial.

In our research, we have used the three assessment protocols that output most information for domain experts. These are called Unilateral Stance (US), Limits of Stability (LOS) and Rhythmic Weight Shift (RWS). The three assessment protocols generate time series showing events, that is, regions of special interest for experts in the domain. The possible events appearing in the time series of each assessment protocol are described below:

- US (*Unilateral Stance*): The aim of this assessment protocol is to measure patient ability to keep their balance standing on one foot with either eyes open and eyes closed. Ideally patients should remain static throughout the assessment protocol and not sway at all. Too much sway is a possible sign of some sensory or vestibular disorder. Little sway would be a good symptom of patient balance.
  Each trial lasts 10 seconds, during which the patient has to remain as immobile as possible with just one foot on the plate (Figure 7).
  To complete the assessment protocol, patients have to perform all four trials (each trial is repeated three times. Depending on the trial, the patient will have their eyes open or closed. The four assessment protocol trials are:
  - Left foot (on platform) and eyes open
  - Left foot (on platform) and eyes shut
  - Right foot (on platform) and eyes open
  - Right foot (on platform) and eyes shut
  In this assessment protocol, the computer stores four integer values every 10 milliseconds, that is, 1,000 tuples each with four values are stored for each repetition of each trial.
  The most important aspect of this assessment protocol from the expert's viewpoint is the analysis of patient balance losses during the assessment protocol. The main aim is to find out how much the patient sways, whether this swaying ends in balance loss, that is, whether the patient is obliged to put down the foot which should be raised throughout.
- LOS (*Limits of Stability*): The aim of this assessment protocol is to measure the maximum distance patients can intentionally displace their centre of gravity and stay there for a time with both feet on the platform without losing balance or stepping. This assessment protocol is useful for measuring patient reaction time, directional control, centre of gravity movement velocity and maximum distance that patients can displace their centre of gravity without moving their feet (Figure 8). Ideally reaction time should be low. A high reaction time is a possible sign of cognitive dysfunction or motor disease. Additionally, patients should also be able to control the direction of movement, which they should do quickly and without hesitation. Low direction of movement control could be sign of the patient having some central nervous system disease, such as Parkinson's disease. Patients should also get as close as possible to targets without moving the position of their feet on the platform. A patient that is unable get close to the target may have motor problems.
  Each assessment protocol trial lasts 10 seconds, during which patients have to try to displace their centre of gravity towards a target position and stay there until the end of the assessment protocol. During this assessment protocol, a circle is visualized on the computer screen. This circle represents patient centre of gravity. A red box is also displayed. This represents the target point. That is, patients know both the position of their centre of gravity and the target point at any time. Patients have to use different inclination movements to displace their centre of gravity towards the target. Patients have to complete each of the eight trials (each trial is repeated once) to complete the assessment protocol. In each trial, patients have to displace their centre of gravity to a particular position (depending on the trial) in order to reach a particular point until the 10 seconds are up. The assessment protocol trials are detailed below:
  - Forward (Figure 9)
  - Forward–Right
  - Right
  - Backward–Right
  - Backward
  - Backward–Left
  - Left
  - Forward–Left
  In this assessment protocol, the computer stores four integer values every 10 milliseconds, that is, 1,000 four-value tuples are stored for each repetition of each trial. According to the criteria extracted from the medical expert, the most important aspect of this assessment protocol is the analysis of approximations to and deviations from the target. Figure 10 shows an example of a patient trajectory for the LOS assessment protocol Right trial. In this case, patients have to displace their centre of gravity to their right. The green colour highlights the positive movements, whereas the negative movements are highlighted in red. In this particular case, the first movement starts at the source and moves towards the target, a second movement moves away from the target, and a third movement, in this case, positive, again moves towards and reaches the target.
- RWS (*Rhythmic Weight Shift*): The aim of this assessment protocol is to quantify patient ability to voluntarily move their centre of gravity laterally from left to right and forward and backward between two targets at various speeds. To do this, the screen displays a circle and a human figure that represents the patient's centre of gravity. The patient has to incline their body from side to side and move their centre of gravity in pursuit of the moving circle. Several lines are also displayed on screen forming a wall. They represent the point beyond which the centre of gravity should not move. The assessment protocol is useful for measuring patient ability to control the direction in which they are moving (directional control), as well as the centre of gravity movement velocity laterally and forward and backward. Ideally directional control should be high, that is, patient movements should to be precise, track the circle and not be overly brusque. High directional control is a sign that the patient is very able to move their centre of gravity laterally or forward and backward. If directional control is low, patients might have some control of movement disorder. Movement velocity should also be adequate, that is, patients should track, but not overtake or lose, the circle. Each trial of this assessment protocol has a different duration. To complete the assessment protocol, patients have to perform each of its six trials (each trial is repeated once). In each trial, patients should displace their centre of gravity laterally or forward and backward (depending on the trial) in pursuit of the moving circle with both feet on the platform. The assessment protocol trials are:
  - Lateral–Slow. Its duration is 18 seconds.
  - Lateral–Medium. Its duration is 12 seconds.
  - Lateral–Fast. Its duration is 6 seconds.
  - Forward/Backward–Slow. Its duration is 18 seconds.
  - Forward/Backward–Medium. Its duration is 12 seconds.
  - Forward/Backward–Fast. Its duration is 6 seconds.

**Figure 11.**
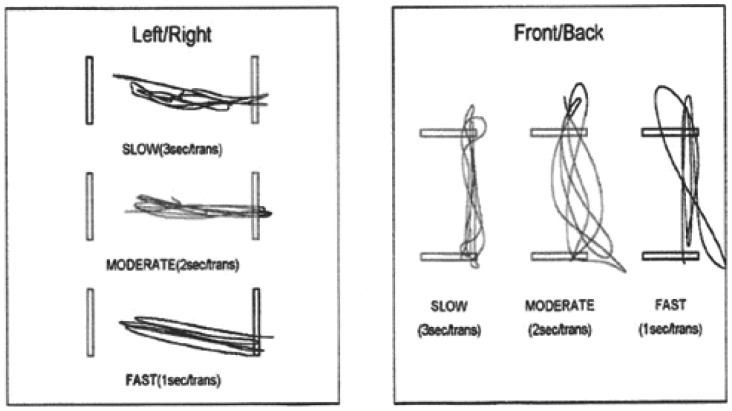
Patient trajectories for the RWS assessment protocol.

Figure 11 shows the patient trajectories for each trial.

In the expert's view, it is interesting to analyse each of the patient transitions from one side to another for this assessment protocol. These transitions should preferably be as smooth as possible and the time series should approximate a sinusoidal curve. This would represent an ideal case of directional control.

### 4.1. Data Selection and Recording

While patients perform the stabilometric assessment protocols, the posturographic platform sends data to a central computer through a data transmission cable. The physician is responsible for giving the patients instructions on how to perform the assessment protocols.

The platform has four sensors, one at each of the four corners: front-right (*FR*), front-left (*FL*), rear-right (*RR*) and rear-left (*RL*). While patients are completing a trial, each of the sensors receives a datum every 10 milliseconds. This datum is the intensity of the pressure that the patient is exerting on the above sensor.

**Figure 12.**
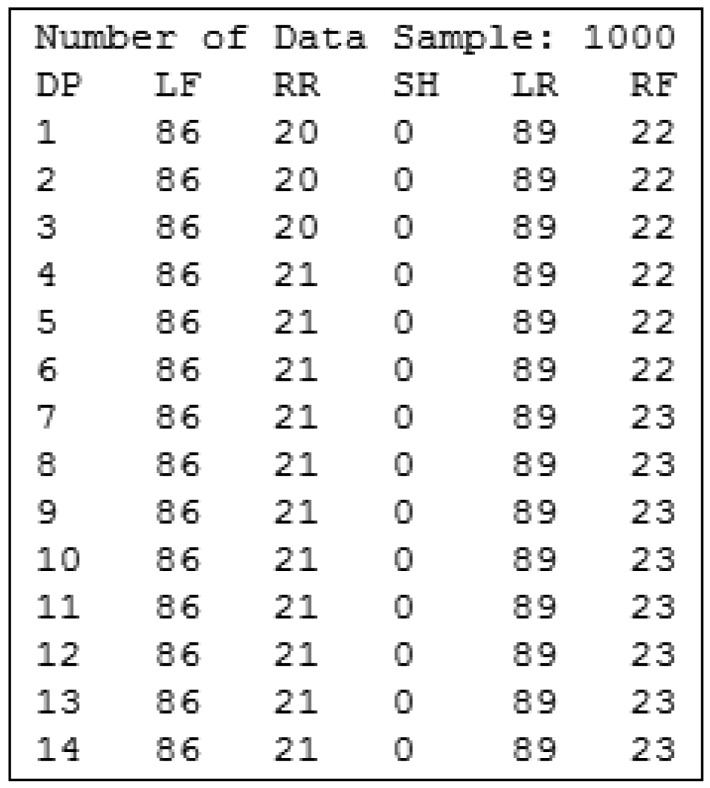
Part of a time series generated by a posturograph.

In short, every 10 milliseconds, the platform sends four integer values to the computer for storage. Therefore, when an assessment protocol is completed, we have a series of values collected at different time points. That is, we have a four-dimensional time series, as four values are stored for each time instant. Figure 12 shows an example of a time series generated by the posturograph, recording, for each time instant (represented as DP in Figure 12), the value of each of the four sensors. The SH value is always 0 as it is only used during the posturograph calibration process.

Once the patient-related time series have been recorded and stored, physicians use the software installed in the computer to visualize and draw conclusions from the data. The software system installed in the computer outputs different parameters about each patient, such as, for example, balance or sway velocity, which are extracted from that patient's time series.

In our research, we used stabilometric time series from a total of 30 patients suffering from Ménière's disease to evaluate the events definition language. Menière's disease affects the internal ear and is primarily characterized by vertigo (code H81.0 of the International Statistical Classification of Diseases and related Health Problems established by the ICD). Stabilometry is primarily concerned with the study of diseases characterized by the presence of vertigo. Thirty is considered a reasonable number of patients, taking into account that access to this type of private information is limited and stabilometric assessment protocols are highly complex (provide a lot of information but are at the same time costly in terms of time and resources).

### 4.2. Events in Stabilometry

We have used the time series events definition language proposed in Section 3 to locate the events in the above time series. We then used the purpose-built software to check the lexical, syntactic and semantic correctness of the events definition for the stabilometric case. Finally, we used this software to translate the events definition to source code in C#. This source code has been used to identify the events in stabilometric time series.

In the following, we present, for each assessment protocol type, the events definition used as a basis for translation and integration into the global system. Events are defined for the US, LOS and RWS assessment protocols only, as they are the only assessment protocols where, according to the criteria elicited from medical experts, it makes sense to speak of events.

#### 4.2.1. US Assessment Protocol

**Figure 13.**
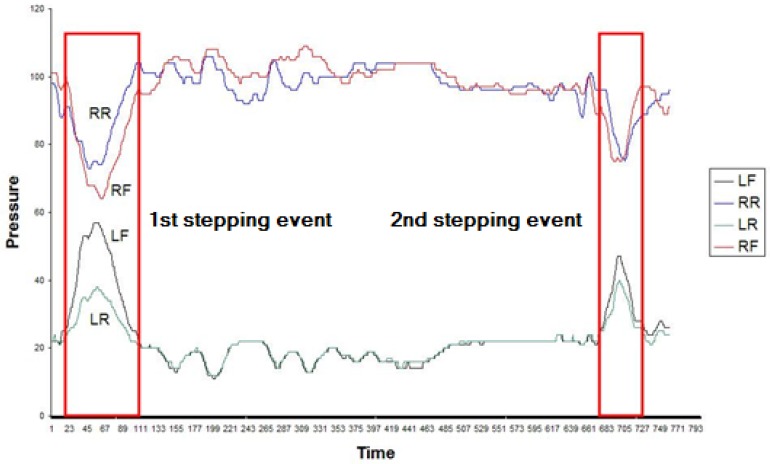
US assessment protocol time series highlighting two stepping events.

The US assessment protocol aims to measure the patient's ability to keep their balance standing on one foot with either eyes open or eyes closed. Ideally patients should remain static and not sway at all throughout the assessment protocol. An interesting event type for this assessment protocol is when patients lose balance and put down their raised foot. This event type is known in the domain as *stepping*. When there is stepping, the raised foot sensor will record the increased pressure. Figure 13 shows the time series for a patient that has completed the US assessment protocol. The curves at the top of the chart are the values recorded by the RR and RF sensors, that is, the right foot sensors, which is the support foot. The curves that at the bottom of the chart plot the values recorded by the LR and LF sensors, that is, the raised left foot sensors. This chart highlights the pressure peaks that are generated when a stepping event takes place.

The first interesting circumstance illustrated in Figure 13 is the fact that the value of the LR and LF dimensions is almost static (with a small margin of variance) throughout, except when stepping takes place. Therefore, a balance value can be defined for those dimensions. This balance value could be a statistical measure like the mode.

Figure 13 also clearly illustrates that every time that there is a stepping event, there is a particular time instant where the LF and LR sensors record a local maximum and the RR and RF sensors record a local minimum. This point is approximately at the centre of the stepping event.

Therefore, the central point of a stepping event could be defined as the point where:

1. There is a local minimum of RR and RF and a local maximum of LF and LR simultaneously.
2. The local maximum of LF is relatively distant from the balance value (mode) of the LF time series.
3. The local maximum LR is relatively distant from the balance value (mode) of the LR time series.

If we locate points that meet the above conditions, we will have located the event. To determine when the event starts and ends, all we have to do is to consider the points where LR and LF intersect with their respective modes and take the point immediately prior to the central point of the stepping event as the start of the point and the point immediately after the central point of the stepping event as the end of the stepping event.

For the time series events definition language to define the above events, it is necessary first to state the four dimensions of which each stabilometric time series is composed (this statement would be preceded by the reserved word *def*).

// Time series statement
lf series;
lr series;
rf series;
rr series;

As mentioned earlier, we are interested in searching for time instants where RF and RR are minimum and LF and LR are maximum. This set of points is called *cand1*:

set cand1
{
x in lf
such that
(x in max(lr))
&& (∃ y in max(lf) such that abs(x-y) < TIME_AXIS_THLD)
&& (∃ z in min(rf) such that abs(x-z) < TIME_AXIS_THLD)
&& (∃ w in min(rr) such that abs(x-w) < TIME_AXIS_THLD)
}; // The TIME_AXIS_THLD is elicited from the expert

However, we are only interested in the points of *cand1* where the value of the series corresponding to the raised foot is relatively distant from the mode. This set of points is *cand2*:

set cand2
{
y in cand1
such that
((lf.value(y) + lr.value(y)) – (mod(lf) + mod(lr))) > INTENSITY_THLD
}; // The INTENSITY_THLD threshold is elicited from the expert

Finally, we need the intersections of one of the two time series for the raised foot (for example, *LF*) with the balance value (mode) in order to then define the start and end of the event. This set of points will be called *intersec*:

set intersec
{
z in lf
such that
lf.value(z) == mod(lf)
};

The next step after defining the sets of necessary points is to define the events. In this case, the break point of the event is a member of *cand1*, whereas the start and end of the event will be members of the *intersec* set. To find out where each event starts and ends, we have to analyse the set of points *cand2*. As specified above, the stepping events would be:

event stepping
{
break_point in cand2,
start in intersec,
end in intersec
such that
(previous(break_point,intersec) == start)
&& (next(break_point,intersec) == end)
};

Clearly, we search for the centre of the stepping event (a point in *cand2*) and then we search for the points that come before and after this point in the *intersec* set, thus perfectly defining the event.

#### 4.2.2. LOS Assessment Protocol

The procedure for the LOS assessment protocol is somewhat different than in the US assessment protocol. Generally speaking, the goal of this assessment protocol is for patients to displace their centre of gravity to a target point and stay there for a time. For the expert, it is especially interesting to analyse the approximations and deviations from the target. These are the two events for definition.

In this case, the four-dimensional stabilometric time series has been transformed into a new one-dimensional time series that records the distance to the target at each time instant. To do this, we have used the values of the four dimensions to determine the position of patient centre of gravity at each time instant and have calculated the distance of the centre of gravity to the target by means of elementary geometric operations. We want to find the parts of the transformed series whose distance to the target increases (deviations) and decreases (approximations).

def{// Time series definition serie transf; // Definition of approximation events Approximation event {  break_point in max(transf),  start in max(transf),  end in min(transf)  such that    (break_point == start)   && (next(break_point,min(transf)) == end) }; // Definition of deviation events move away event {  break_point in min(transf),   start in min(transf),   end in max(transf)   such that    (break_point == start)    && (next(break_point,max(transf)) == end) };}

Note that approximations are the parts between one time series maximum and the next minimum. Deviations, on the other hand, are the parts between a time series minimum and the next maximum.

#### 4.2.3. RWS Assessment Protocol

In the RWS assessment protocol, we work with the four-dimensional time series. In this assessment protocol, the dimensions are paired, that is, they follow a similar pairwise trajectory. The trajectory also has a sinusoidal form, such that when two of the dimensions are maximum, the other two are minimum. Additionally, there are points where four dimensions of the series intersect.

The events or segments of interest stretch from the points where RR and RF are minimum and LF and LR are maximum to the points where RR and RF are maximum and LF and LR are minimum, taking in a point where the four time series intersect. In the following we present the proposed definition for this type of event:

def{ // Time series definition serie lf; serie lr; serie rf; serie rr; // Complex sets definition // Points at which the four time series // intersect set cand1 {  x in lf  such that   (abs(lf.value(x) - lr.value(x)) < THLD)   && (abs(lf.value(x) - rf.value(x)) < THLD)   && (abs(lf.value(x) - rr.value(x)) < THLD) }; // The THLD is elicited from the expert // Those points where there LF and LR are maximum and RF //and RR are minimum // in set cand2 {  y in lf  such that   (y in max(lf))   && (∃ x in max(lr) such that abs(y-x)< THLD′)   && (∃ w in min(rf) such that abs(y-w) < THLD′)   && (∃ z in min(rr) such that abs(y-z) > THLD′) }; // The THLD′ threshold is elicited from the expert // Those points where LF and LR are minimum and RF //and RR are maximum set cand3 {  z in lf  such that   (z in min(lf))   && ((∃ x in min(lr) such that abs(z-x) < THLD″)   && (((∃ y in max(rf) such that abs(z-y) < THLD″)   && (((∃ w in max(rr) such that abs(z-w) < THLD″) }; // The THLD″ threshold is elicited from the expert // Events definition // The events range from one point of cand2 to another of cand3, // through one of cand1 lateral event {  break_point in cand1,  start in cand2,  end in cand3   such that     (previous(break_point, cand2) == start)    && (next(end,cand1) == break_point) };}

We can use the proposed translator to obtain source code in a high-level language from this events definition without having to make low-level changes in the application.

### 4.3. Discussion of Results

One of the main motivations for the proposed events definition language is that existing methods for identifying events in time series, which are closely associated with particular domains, are extremely specialized and require major very low-level changes for adaptation to other domains. Therefore, one of the biggest advantages of the language proposed in this article is its generality and applicability to multiple domains.

Thanks to the implemented translator, the events definition language should be able to generate source code in a high-level language in order to precisely locate events in time series, emulating a domain expert. In this respect, our proposal can be considered as an intelligent expert system as it emulates the reasoning of experts in a particular domain and it is intended to be used by those experts.

To evaluate our proposal, we have taken stabilometric data from a total of 30 patients, each of which completed different assessment protocol trials. Additionally, the medical protocol stipulates that each trial of certain should be repeated three times. The US assessment protocol has four, the LOS assessment protocol has eight and, finally, the RWS has six trials. The total number of time series considered is, therefore, 1,620 = 30(patients) × 18(trials) × 3(repetitions).

To evaluate the precision of our events identification proposal on the above 1,620 time series, we asked a stabilometric domain expert to identify the events in those series and we then applied our technique to do the same thing. For each time series, we have measured the precision of our proposal using Equation (1) that measures the degree of similarity (*SIM_Exp_Lang*) between the number of events identified by the expert (*#EV_Exp_*) and by our language (*#Ev_Lang_*). Note that this formula offers a normalized result in the interval [0,1], where 1 indicates a total coincidence between the number of events identified by the expert and by the language. The worst case is when the expert locates events in a series and our system does not identify any:

(1) $${\text{SIM}}_{{\text{Exp}}_{\text{Lang}}}=1-\frac{\left|\#Ev_{\text{Exp}}-\#Ev_{\text{Lang}}\right|}{\#Ev_{\text{Exp}}}.$$

From a global analysis of the 1,620 time series, we find that there is good match between the expert and proposed language, as shown by the mean similarity, which is greater than 98%, between the expert and language (Table 9). This value is very close to the ideal.

Apart from the global results, we have conducted a detailed analysis of the time series. To do this, we have taken a representative sample of the situations where the language turned out to be worse: 60 time series where the expert and language were least similar, as shown in Table 10. This table lists the series identifier, the assessment protocol to which it belongs (US, LOS or RWS), the trial, number of events identified by the expert, number of events identified using the language and the match between the two. For simplicity's sake, the trials have been encoded in the table as established in [33] by the company that commercializes the posturograph used in this research.

To explain the behaviour of the *SIM_Exp_Lang* in these time series, we have studied the descriptive statistics of the *SIM_Exp_Lang* variable, shown in Table 11.

Fitting the *SIM_Exp_Lang* variable to a probability law, we find that the best fit of that variable is equivalent to a normal distribution. The Kolmogorov-Smirnov test data prove this point for that fit, as shown in Table 12.

**Figure 14.**
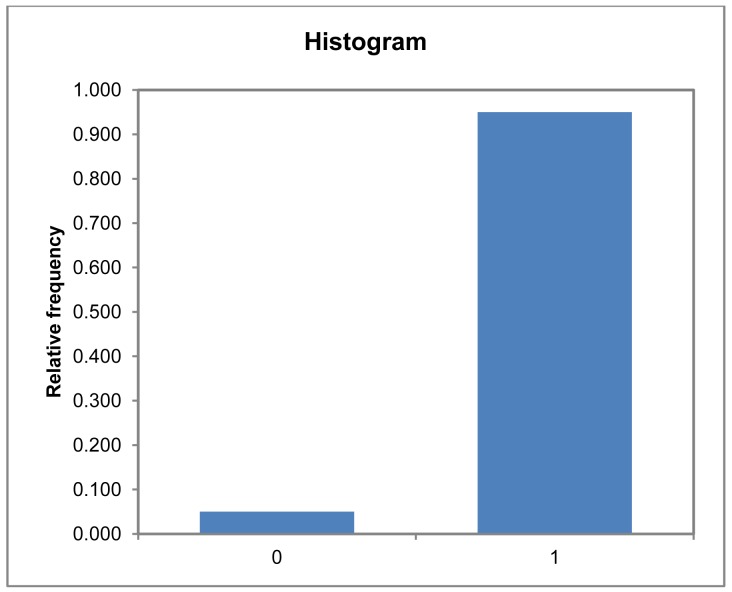
Discretized histogram of the SIM_Exp_Lang variable.

At the significance level alpha = 0.050, we can accept the null hypothesis that there is no difference between the accumulated empirical and theoretical distributions of a normal and of the *SIM_Exp_Lang* variable on which the test is based. Looking at the discretized histogram of the *SIM_Exp_Lang* variable, we obtain the result shown in Figure 14.

We find that, even in the 60 worst cases, the system responds with a *SIM_Exp_Lang* very close to 1 in over 90% of the cases. Having characterized the variable under study, we proceed to study the covariance of the *SIM_Exp_Lang* variable with respect to the characteristics studied in the sample, assuming that each variable is explained in terms of the other qualitative variables (*assessment protocol* and *trial*) and quantitative variables (*#Ev_Exp_*). To do this, we conduct an ANCOVA of the *SIM_Exp_Lang* variable with the adjustment results shown in Table 13.

The adjusted value R^2^ indicates that 52% of the *SIM_Exp_Lang* variable is explained by the variables that characterize each time series. From these data, we deduce that system behaviour is independent and equivalent in each assessment protocol and trial type. The information value generated by such variables is not very representative, as indicated by Fisher's F equal to 5.297, which is much greater than the critical F value for the degrees of freedom of *SIM_Exp_Lang* (100) and the degrees of freedom of the studied variables 815), which, with an alpha value = 0.05, is F = 2.123.

**Figure 15.**
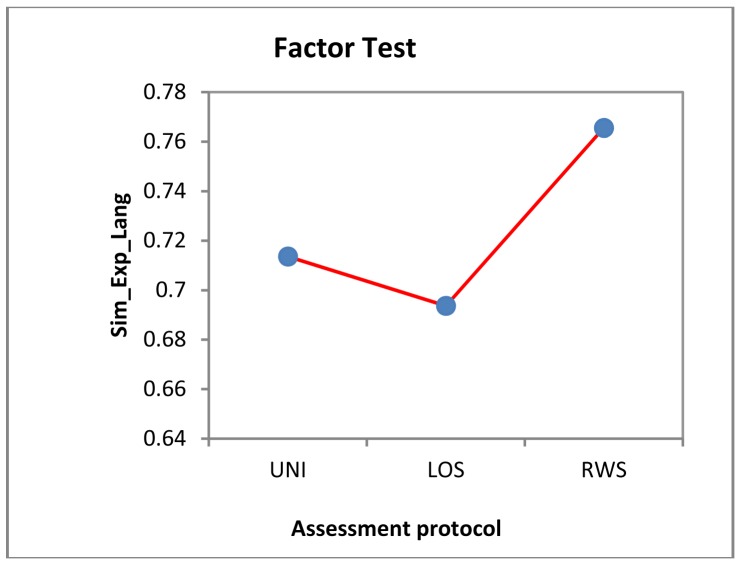
Relationship between SIM_Exp_Lang and the Assessment protocol variable.

**Figure 16.**
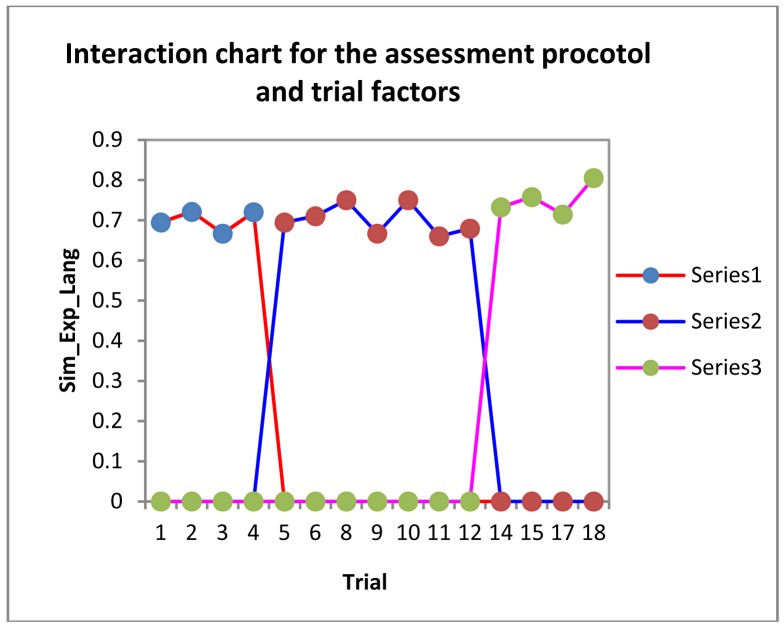
Relationship between SIM_Exp_Lang and the trial variable.

Considering the information that each variable inputs to the model, we have that the number of events identified by the expert is irrelevant for *SIM_Exp_Lang* (F = 35.185), as is the assessment protocol type (F = 10.89). The only variable that may input some information to the above regression model is the particular trial completed as part of the assessment protocol. To visualize how such variables affect *SIM_Exp_Lang*, we have plotted the charts illustrated in Figures 15 and 16.

This chart shows the mean of *SIM_Exp_Lang* obtained in each assessment protocol type. The worst reading is for the LOS assessment protocol, whereas the best is for the RWS assessment protocol. After analysis, it appears that:

- The deviations for the US assessment protocol occurred when stepping took place towards the end of the assessment protocol and patients did not complete the assessment protocol because it ended before they regained their balance. This is a circumstance that the expert identifies by looking at the series, but the language does not identify as the specified events definition conditions are not met, namely, that the event must have a measurable extreme point within the series and not a point beyond the end of the series that has to be guesstimated. The fuzzy consideration of this type of events is beyond the scope of this research and is stated as future research in Section 5.
- The deviations for the LOS and RWS assessment protocols are due to the fact that the expert confuses overlapping trajectories and considers different trajectories to be the same. This means that our system identifies events that are not visible to the expert and is a very valuable support tool in these cases.

Precisely, the results obtained for the LOS assessment protocol were worse because the above discrepancy occurred more often in the LOS assessment protocol time series. The RWS assessment protocol results were better because the discrepancy is less common in the RWS assessment protocol time series.

Looking at how each particular assessment protocol affects the value obtained in *SIM_Exp_Lang*, we get the chart illustrated in Figure 16.

In this chart, we find that the mean value for *SIM_Exp_Lang* never drops below 7 for any of the 60 worst time series. The worst ratings are for trials 3, 9 and 11, because the resulting time series were affected by the above discrepancy, which was compounded by the fact that the variable (*SIM_Exp_Lang*) formula penalizes such discrepancies especially when there are relatively few events, as in the case of the time series of trials 3, 9 and 11.

The next step is to check whether the system has operated equivalently with respect to each patient type without any variables characterizing patients possibly having influenced the system. To do this, we ran a study of the variances obtained for the value *SIM_Exp_Lang* in each of the time series for the same patients. An ANOVA study shows that there are significant variations in the value of *SIM_Exp_Lang* from one patient to another. The results of the study are shown in Table 14.

We have run two types of study, called type I and type III sums of squares, to check whether the data are balanced, that is, whether the system behaves equally with respect to one and the same time series irrespective of the processing order. The value is equal in both studies and additionally Fisher's F is also very high, F = 828 ≫ 253, where 253 is the critical F for alpha = 0.05 for those degrees of freedom. Accordingly, the system is 99.999% sure to have behaved equally with respect to different patients, irrespective of their particular characteristics.

After a detailed study of the *SIM_Exp_Lang*, the last part of the study is given over to the #Ev_Exp_ variable, that is, the number of events that the system identifies, which is evidently a causal variable of the values obtained for *SIM_Exp_Lang*. The aim of this study is to obtain a reliable linear or parametric regression model with the aim of inferring how many events the system will identify in terms of the events identified in the same time series by an expert. To do this, we built a regression model that we used to obtain the data shown in Table 15.

This time the resulting regression model is very reliable, with an adjusted R^2^ very close to 1. This means that the value of Ev_Lang_ is totally dependent on Ev_Exp_, something which is, on the other hand, to be expected, and can be inferred from the modelling shown in Table 16.

**Figure 17.**
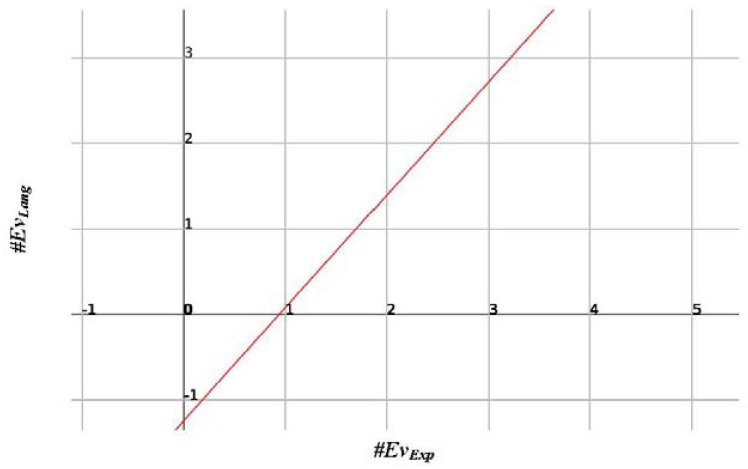
Regression model between variables *Ev_Exp_* and *Ev_Lang_*.

The model is described by Equation (2) that is illustrated in Figure 17.

(2) #EvLang=−1.24023586101952+1.32044128835912∗#EvExp

**Figure 18.**
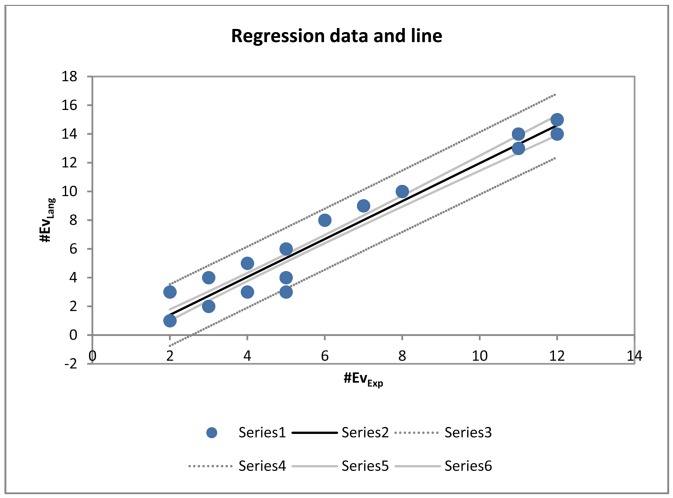
Regression data and straight line between variables *Ev_Exp_* and *Ev_Lang_*.

Using that model, based on the experience obtained for the 60 worst cases, regression yields the number of events identified by the system in a time series with the precision plotted by the chart in Figure 18.

Analysing the regression line, we find that, considering that the model is discrete and deterministic, the system successful and accurately elicits the events, as if it were an expert, for time series with between zero and eight events. As of eight events, the model provides an equivalent linear deviation to the model equation. This model could be used, as described in future research lines, to adjust the system results by weighting its model with fuzzy logic. In sum, for the 1,620 series, even the 60 with which the model behaved worse, the model is adjusted to the results that an expert would obtain provided that the series does not contain more than eight to ten events. This is unlikely to be the case for time series in the stabilometric domain (as shown in Table 9, where $\bar{\#Ev_{\text{Exp}}}=5.32$) and any other domain, taking into account the duration of the analysed time series, which is of the order of a few seconds.

Another important point apart from precision is to evaluate the generality of the proposed language. In our research, we have tested the language on disparate domains. One is stabilometry, considered in this paper as a reference domain. However, our language has been applied to other branches of medicine, such as neurology and cardiology, to define events in time series extracted from electroencephalograms and electrocardiograms, respectively. Additionally, the language has been applied to define events in stock exchange time series where the points of interest of the series occur when there is sharp drop in value (there is a good selling opportunity just before the fall) or a sharp increase (there is a good buying opportunity just before the increase). For an exhaustive description of the application of the language to the above domains and the respective results, see [13].

## 5. Conclusions and Future Work

There are many proposals in the area of time series analysis. However, there are new challenges in the field, one of which is the identification of regions of interest (events) for experts in each domain.

We have proposed a one-dimensional and multi-dimensional time series events definition language. Additionally, the specification of the events definition language has been rounded out with the development of a translator that checks whether the events definition is lexically, syntactically and semantically correct. If so, that events definition is automatically translated to source code of a high-level programming language. The proposal of this language is a big help and saves time for its potential users, as time series events identification is a complex task requiring costly ad hoc methods for each domain.

The proposed language has been tested on data from the medical domain with satisfactory results. Specifically, the techniques have been applied to structurally complex data from the field of stabilometry, which is a discipline that studies human balance. The good results obtained when testing the events definition language on the data of different stabilometric assessments confirm its generality.

Some research lines raising new challenges to upcoming researchers remain open. An important aspect related to events definition is how to determine the conditions that decide whether something is or is not an event in a particular domain. In the case of the domain studied in this research, we have defined the events using relational and threshold operators. For example, in the stabilometric domain US assessment protocol, a region of a series is considered to be a stepping event when there is a local extreme point and the stepping intensity is greater than a particular threshold. If pressure is less than the threshold, it is not considered a stepping event. In many other domains, however, it is very difficult, if not impossible, to make such a distinction. Therefore, we fancy fuzzy logic as being an interesting and, at the same time, useful option for tackling this line of research. This way, it would be possible to incorporate the concept of event *certainty* into the proposed schema. This would indicate how sure a time series region is to be an event. Thanks to the concept of certainty, we would obtain richer events models in which more importance would be attached to events with greater certainty.

Another important matter related to events definition is that domain experts may not be computer literate enough to be able to program an events definition alone and, therefore, may require help from a computer specialist to do this. To overcome this difficulty, the proposed schema could incorporate a functionality enabling experts to define the events visually without having to use the language directly. This functionality could be composed of a layer to automatically transform the expert's graphical definition into a textual events definition based on the proposed language. Work on this topic is already under way.

## Figures and Tables

**Table 1.** Basic events definition language elements.

**Basic Elements**	**Description**	
Time series	Definition	Ordered set of measurements in a time interval., Dimensions are conceived as interdependent mathematical functions in multidimensional time series
	Examples	stockexchange_price **series**;
Statistical measures	Definition	A series of basic predefined statistical measures is defined for each dimension
	Examples	Average (**avg**), mode (**mode**), median(**med**), standard deviation (**stdev**) and variance (**var**)
Sets of predefined points	Definition	Some points tend to be of interest in most domains, like, for example, the moments in the series where a maximum is reached
	Examples	Series timestamps (**timestamps**), local maxima (**max**) and local minima(**min**)
Arithmetical operators	Definition	Basic arithmetical operators
	Examples	+, −, *, /, *etc.*
Relational operators	Definition	Operators that are useful for comparing and establishing relationships among elements
	Examples	<, >, <=, *etc.*
Arithmetical functions	Definition	More sophisticated arithmetical functions
	Examples	Square root (**sqrt**), exponent (**exp**), absolute value (**abs**), *etc.*
/…/	/…/	
Logic operators	Definition	Operators that can be applied to Boolean type data
	Examples	**AND, OR, NOT**
Set operators	Definition	Operators for handling sets
	Examples	Ø, ∩, ∪, ⊃, ⊇, ⊂, ⊆, ∀, ∃, *etc.*

**Table 2.** Event definition language syntax.

**Events Definition Parts**		**Description**	
Time Series		Syntax	**series** series_name;
		Examples	**series** series_1_; **series** series_2_;
Sets of points of interest	Using conditions	Syntax	**set** set_name { id_1_ **in** set_name′ **such that** CONDITION };
		Example	**set** MaxMultiples50 **{**x **in max(**ST_A_**) such that ((**x **%** 50**) ==** 0**)}**;
/…/		/…/	
Sets of points of interest	Using operators	Syntax	**set** new_set **=** old_set_1_ **OPERATOR** old_set_2_;
		Examples	**set** mySet **=** mySet_1_ **joint** mySet_2_; **set** mySet′ **=** mySet_1_ **diff** mySet_2_; **set** mySet″ **=** mySet_1_ **intersec** mySet_2_;
Events		Syntax	**event** event_name **{ break_point in** set_name, **start in** set_name_1_, **end in** set_name_2_ **such that** CONDITION **}**
		Example	**event** ev **{ break_point in** MaxMultiples50, **start in** TS_A_, **end in** TS_A_ **such that** **previous(break_point**, TS_A_**)** == **start** **&& next(break_point**, TS_A_**)** == **end }**;

**Table 3.** Arithmetic operators.

**AOP**	**+**	**-**	*	**/**	**%**	**abs**	**exp**	**sqrt**
n	0	1	2	3	4	5	6	7

**Table 4.** Relational operators.

**ROP**	**==**	**!=**	**<**	**<=**	**>**	**>=**
n	0	1	2	3	4	5

**Table 5.** Conditional operators.

**CMDOP**	**&&**	**‖**	**!**
N	0	1	2

**Table 6.** Time series access operators.

**TSAOP**	**.value**	**f′**	**f″**
N	0	1	2

**Table 7.** Set operators.

**SETOP**	**n**
=	0
in	1
joint	2
intersec	3
diff	4
subc	5
subcp	6
sup	7
supcp	8
exists	9
forall	10
isfirst	11
islast	12
next	13
previous	14

**Table 8.** Syntactic analyser grammar.

**Grammar Elements**	
**Axiom**	A
**Terminals**	{def, {, }, serie, id, ; , ‘,’ , (, ), mean, mode, median, stdev, variance, max, min, set, in, event, such, that, ‖, &&, !, true, false, <=, <, >=, >, ==, !=, .value, f′, f″, +, −, *, /, %, abs, exp, sqrt, integer_cst, real_cst, break_point, start, end, =, joint, intersec, diff, subc, subcp, supc, supcp, exists, forall, isfirst, islast, next, previous, int, bool, float, void, extmethod, function}
**Non Terminals**	{A, D, ST, ST_1_, STAT_TYPE, BS_TYPE, S, CJ, OP_CJ, EV, EV_1_, SING, BG, ED, C, C_1_, C_2_, C_3_, R, E, E_1_, E_2_, E_3_, EM, P, P_1_, TD, TPS, TPS_1_, TP}
**Productions**	A 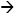 def { D } | λ D 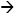 EM ST S EV ST 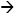 serie id; ST_1_ ST_1_ 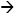 serie id; ST_1_ | λ S 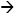 set id {CJ}; S | set id = id OP_CJ id; S | λ CJ 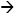 id in id such that C OP_CJ 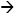 joint | intersec | diff EV 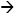 event id {SING BG ED such that C}; EV_1_ EV_1_ 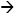 event id {SING BG ED such that C}; EV_1_ | λ SING 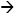 break_point in id, BG 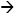 start in id, ED 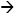 end in id C 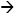 C ‖ C_1_ | C_1_ C_1_ 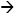 C_1_ && C_2_ | C_2_ C_2_ 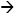 !C_3_ | C_3_ C_3_ 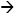 (C) | true | false | R R 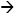 E <= E | E < E | E >= E | E > E | E == E | E != E | E in id |subc (id,id) | subcp(id,id) | supc(id,id) | supcp(id,id) | exists id in id such that C | forall id in id such that C | isfirst(E,id) | islast(E,id) E 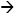 E + E_1_ | E – E_1_ | E_1_ E_1_ 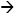 E_1_* E_2_ | E_1_/E_2_ | E_1_%E_2_ | E_2_ E_2_ 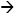 -E_2_ | E_3_ E_3_ 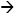 id | integer_cst | real_cst | start | break_point | end | id.value(E) | f′ id(E) | f″ | id(E) | next(E,id) | previous(E,id) | abs(E) | sqrt(E) | exp(E,E) | (E) | BS_TYPE(id) | STAT_TYPE (id) | id (P) BS_TYPE 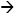 max | min STAT_TYPE 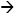 mean | mode | median | stdev | variance P 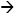 E P_1_ | λ P_1_ 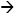 , E P_1_ | λ EM 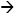 extmethod id TD integer_cst TPS function ; EM | λ TD 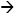 int | float | bool | void TPS 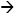 TP TPS_1_ | λ TPS_1_ 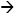 , TP TPS_1_ | λ TP 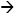 int | float | bool

**Table 9.** Global results of the application of the events definition language to the stabilometric domain.

***#Series***	$\bar{\#{\text{Ev}}_{\text{Exp}}}$	$\bar{\#{\text{Ev}}_{\text{Lang}}}$	$\bar{\text{SIM\_Exp\_Lang}}$
1620	5.32	5.64	0.981

**Table 10.** Detailed results of the 60 least similar stabilometric time series.

***Series_Id***	***Assessment Protocol***	***Trial***	***#Ev****_Exp_*	***#Ev****_Lang_*	***SIM_Exp_Lang***
1	US	1	3	2	0.67
2	US	1	4	3	0.75
3	US	1	3	2	0.67
4	US	2	5	4	0.80
5	US	2	2	1	0.50
6	US	2	5	4	0.80
7	US	2	4	3	0.75
8	US	2	4	3	0.75
9	US	2	3	2	0.67
10	US	2	4	3	0.75
11	US	2	3	2	0.67
12	US	2	4	3	0.75
13	US	2	5	4	0.80
14	US	2	4	3	0.75
15	US	2	3	2	0.67
16	US	3	3	2	0.67
17	US	3	3	2	0.67
18	US	4	5	4	0.80
19	US	4	4	3	0.75
20	US	4	3	2	0.67
21	US	4	5	3	0.60
22	US	4	4	3	0.75
23	US	4	3	2	0.67
24	US	4	4	3	0.75
25	US	4	5	4	0.80
26	US	4	3	2	0.67
27	US	4	4	3	0.75
28	LOS	5	3	4	0.67
29	LOS	5	4	5	0.75
30	LOS	5	3	4	0.67
31	LOS	6	6	8	0.67
32	LOS	6	3	4	0.67
33	LOS	6	4	5	0.75
34	LOS	6	5	6	0.80
35	LOS	6	3	4	0.67
36	LOS	8	4	5	0.75
37	LOS	8	4	5	0.75
38	LOS	9	3	4	0.67
39	LOS	10	4	5	0.75
40	LOS	11	3	4	0.67
41	LOS	11	2	3	0.50
42	LOS	11	3	4	0.67
43	LOS	11	5	6	0.80
44	LOS	11	3	4	0.67
45	LOS	12	4	5	0.75
46	LOS	12	5	6	0.80
47	LOS	12	3	4	0.67
48	LOS	12	2	3	0.50
49	RWS	14	7	9	0.71
50	RWS	14	8	10	0.75
51	RWS	15	11	14	0.73
52	RWS	15	12	15	0.75
53	RWS	15	11	14	0.73
54	RWS	15	12	15	0.75
55	RWS	15	12	14	0.83
56	RWS	17	7	9	0.71
57	RWS	18	11	13	0.82
58	RWS	18	12	14	0.83
59	RWS	18	11	13	0.82
60	RWS	18	12	15	0.75

**Table 11.** Descriptive Statistics of the *SIM_Exp_Lang*.

	**N**	**Mean**	**Std. Dev.**	**Variance**	**95% Confidence Interval for Mean**		**Minimum**	**Maximum**

					**Lower Bound**	**Upper Bound**		
*SIM_Exp_Lang*	60	0.717	0.074	0.006	0.698	0.736	0.5	0.833

**Table 12.** Kolmogorov-Smirnov Test data fitting *SIM_Exp_Lang*.

D	0.204
D (standardized)	1.579
p-value bilateral	0.054
alpha	0.05

**Table 13.** ANCOVA of the *SIM_Exp_Lang* variable.

		
	**Adjustment Coefficients:**				
		
	R (correlation coefficient)			0.802	
	R^2^ (coefficient of determination)			0.644	
	R^2^aj. (adjusted coefficient of determination)			0.522	
	SCR			0.118	
		

**Evaluation of the Value of the Information Output by the Variables (H0 = Y = Moy(Y)):**					

**Source**	**df**	**Sum of Squares**	**Mean Square**	**Fisher's F**	**Pr > F**

Model	15	0.213	0.014	5.297	<0.0001
Residuals	44	0.118	0.003		
Total	59	0.330			
#Ev_Exp_	1	0.094	0.094	35.185	<0.0001
Assessment protocol	2	0.058	0.029	10.829	0.000
Trial	14	0.061	0.004	1.615	0.113

**Table 14.** ANOVA of the *SIM_Exp_Lang* variable.

**Model analysis (Type I SS):**					
**Source**	**DF**	**Sum of Squares**	**Mean Square**	**Fisher's F**	**Pr > F**
#Ev_Exp_	1	916.650	916.650	828.131	<0.0001
**Model analysis (Type III SS):**					
**Source**	**DF**	**Sum of Squares**	**Mean Square**	**Fisher's F**	**Pr > F**
#Ev_Exp_	1	916.650	916.650	828.131	<0.0001

**Table 15.** Regression Model for #Ev_Exp_ variable.

**Summary for Quantitative Variables:**		

**Variable**	**Mean**	**Standard Deviation**
#Ev_Exp_	5.067	2.985
**Adjustment Coefficients:**		

R (correlation coefficient)		0.967
R^2^ (coefficient of determination)		0.935
R^2^aj. (adjusted coefficient of determination)		0.933
SCR		64.200

**Table 16.** Adjustment data of regression model.

**Model Parameters:**						

**Parameter**	**Value**	**Standard Deviation**	**Student's t**	**Pr > t**	**Lower Bound 95%**	**Upper Bound 95%**
Intersection	−1.240	0.269	−4.606	<0.0001	−1.779	−0.701
#Ev_Exp_	1.320	0.046	28.777	<0.0001	1.229	1.412
